# Wheat amylase/trypsin inhibitors (ATIs): occurrence, function and health aspects

**DOI:** 10.1007/s00394-022-02841-y

**Published:** 2022-03-02

**Authors:** Sabrina Geisslitz, Peter Weegels, Peter Shewry, Victor Zevallos, Stefania Masci, Mark Sorrells, Armando Gregorini, Mariastella Colomba, Daisy Jonkers, Xin Huang, Roberto De Giorgio, Giacomo P. Caio, Stefano D’Amico, Colette Larré, Fred Brouns

**Affiliations:** 1 Karlsruhe Institute of Technology (KIT), Karlsruhe, Germany; 2 Netherlands and European Bakery Innovation Centre, Sonneveld Group BV, Papendrecht, and Wageningen University and Research, Wageningen, Netherlands; 3 Laboratory of Food Chemistry, Rothamsted Research, Harpenden, UK; 4 Nutrition and Food Research Group, Department of Applied and Health Sciences, University of Northumbria, Newcastle Upon Tyne, UK; 5 Department of Agricultural and Forestry Sciences, University of Tuscia, Tuscia, Italy; 6 School of Integrative Plant Science, Plant Breeding and Genetics Section, Cornell University, Ithaca, USA; 7 Department of Biomolecular Sciences, University of Urbino “Carlo Bo”, Urbino, Italy; 8 Division of Gastroenterology-Hepatology, Department Internal Medicine, School for Nutrition and Translational Research in Metabolism Maastricht, Maastricht University, Maastricht, Netherlands; 9 Department of Food and Nutrition, Faculty of Agriculture and Forestry, University of Helsinki, Helsinki, Finland; 10 Department of Translational Medicine, St. Anna Hospital, University of Ferrara, Ferrara, Italy; 11 Department of Morphology, Surgery and Experimental Medicine, University of Ferrara, Ferrara, Italy; 12 Institute for Animal Nutrition and Feed, AGES - Austrian Agency for Health and Food Safety, Vienna, Austria; 13 INRAE UR1268 BIA, Impasse Thérèse Bertrand-Fontaine, 44000 Nantes, France; 14 Department of Human Biology, School for Nutrition and Translational Research in Metabolism Maastricht, Maastricht University, Maastricht, Netherlands

**Keywords:** Amylase/trypsin inhibitors, ATIs, Coeliac disease, Wheat allergy, Non-coeliac wheat sensitivity, Intestinal symptoms

## Abstract

Amylase/trypsin inhibitors (ATIs) are widely consumed in cereal-based foods and have been implicated in adverse reactions to wheat exposure, such as respiratory and food allergy, and intestinal responses associated with coeliac disease and non-coeliac wheat sensitivity. ATIs occur in multiple isoforms which differ in the amounts present in different types of wheat (including ancient and modern ones). Measuring ATIs and their isoforms is an analytical challenge as is their isolation for use in studies addressing their potential effects on the human body. ATI isoforms differ in their spectrum of bioactive effects in the human gastrointestinal (GI), which may include enzyme inhibition, inflammation and immune responses and of which much is not known. Similarly, although modifications during food processing (exposure to heat, moisture, salt, acid, fermentation) may affect their structure and activity as shown in vitro, it is important to relate these changes to effects that may present in the GI tract. Finally, much of our knowledge of their potential biological effects is based on studies in vitro and in animal models. Validation by human studies using processed foods as commonly consumed is warranted. We conclude that more detailed understanding of these factors may allow the effects of ATIs on human health to be better understood and when possible, to be ameliorated, for example by innovative food processing. We therefore review in short our current knowledge of these proteins, focusing on features which relate to their biological activity and identifying gaps in our knowledge and research priorities.

## Introduction: what are ATIs?

Amylase/trypsin inhibitors (ATIs) are a group of proteins that are present in the seeds of all cereals (including wheat, barley, rye, maize, millet and rice) and are the most abundant proteins in the water soluble (albumin) fraction of wheat. ATIs inhibit the activities of nutrient degrading enzymes: α-amylase (involved in starch degradation) and trypsin (involved in protein degradation) and are one of several groups of wheat seed proteins which contribute to the natural defence against pests and pathogens.

**Fig. 1:**
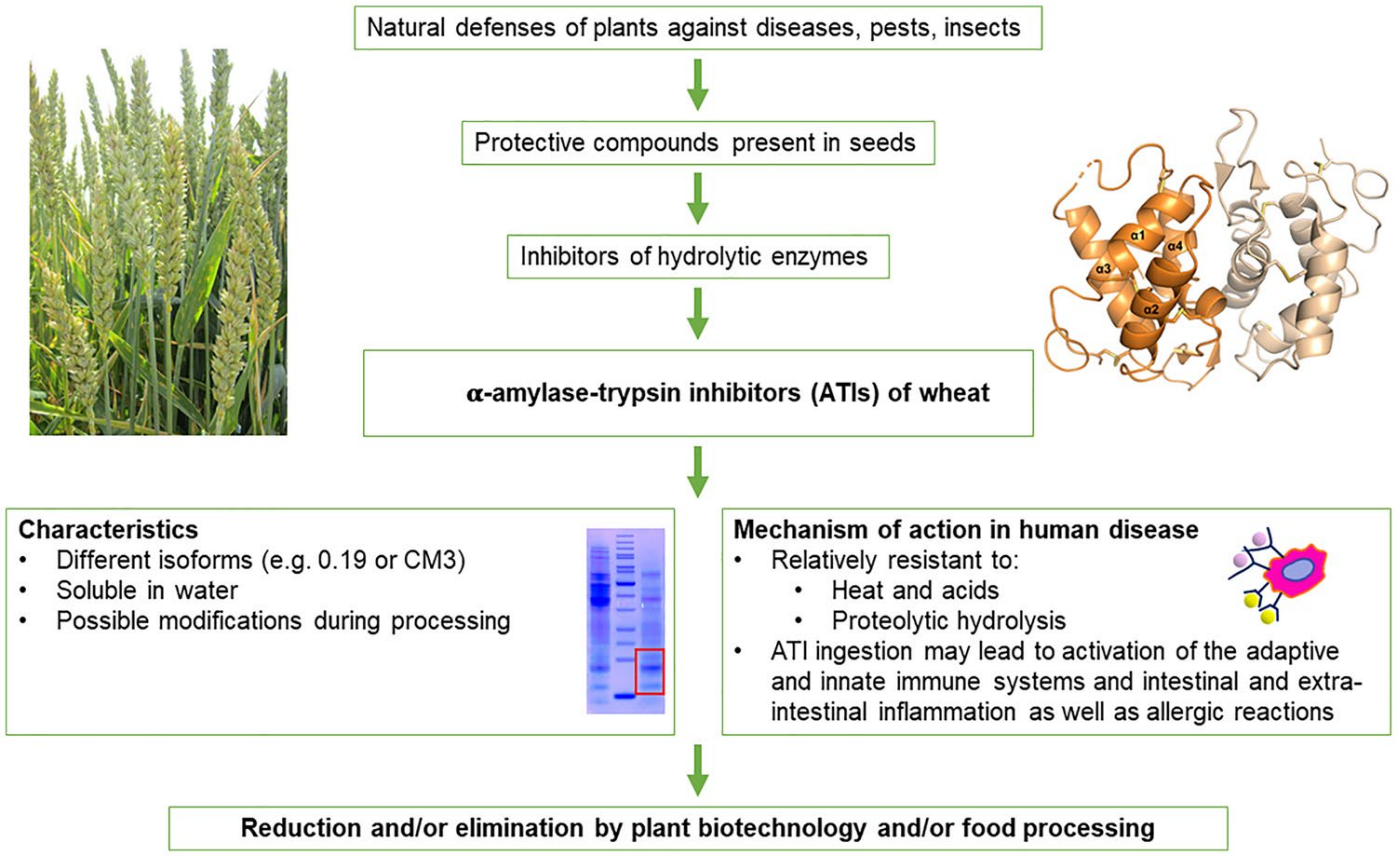
Schematic depiction of key aspects of ATIs in grains and their potential biological effects

Wheat ATIs comprise a group of highly similar proteins (called isoforms), including forms which are active as monomers and dimers and are named 0.19, 0.28 and 0.53 based on their electrophoretic mobility and as tetramers which are soluble in chloroform:methanol (CM) mixtures and also called CM proteins. ATIs differ in their activity against amylases from mammals (including human salivary and pancreatic amylases) and from different types of insects (*e.g*. beetles and larvae of moths) but do not inhibit the endogenous amylases that are present in the wheat seeds, supporting a role in plant defence. A brief overview is presented in Fig. 1.

ATIs were first studied in the 1940s [1] and have been recently critically reviewed [2]. They are well characterised as allergens, particularly in bakers’ asthma (a respiratory allergy to the inhalation of wheat flour) and bakers’ eczema, but also in food allergy to wheat [3, 4]. Recently, there has been renewed interest in ATIs because they have been suggested to play a role in the promotion of two other wheat-related adverse effects: coeliac disease and non-coeliac wheat sensitivity (NCWS, see below). Furthermore, the variation in the inhibitory activities of ATIs suggests that the activity in humans may vary between isoforms [5, 6].

We therefore summarise our current knowledge of the distribution and properties of ATIs in wheat in relation to perceived and established impacts on health and disease.

### Presence and function of ATIs in grains

Most of wheat grown globally is hexaploid wheat (*Triticum aestivum* L., also known as ‘bread wheat’, ‘common wheat’ or ‘soft wheat’), with about 5% being tetraploid durum wheat (*Triticum durum*, also known as ‘pasta wheat’ or ‘hard wheat’). Hexaploid wheat and durum wheats have been produced by intensive breeding, particularly over the last 60 years, and it has been suggested in the scientific, popular and social media that the emphasis of modern breeding on increasing yield, improving resistances to fungal pathogens and improving processing quality has resulted in decreased nutritional quality and increased contents of natural plant protective components, including ATIs. Similarly, it has been suggested that older types of wheat (often called ‘ancient’ and ‘heritage’ wheats) may be expected to contain lower amounts of ATIs [7].

**Fig. 2:**
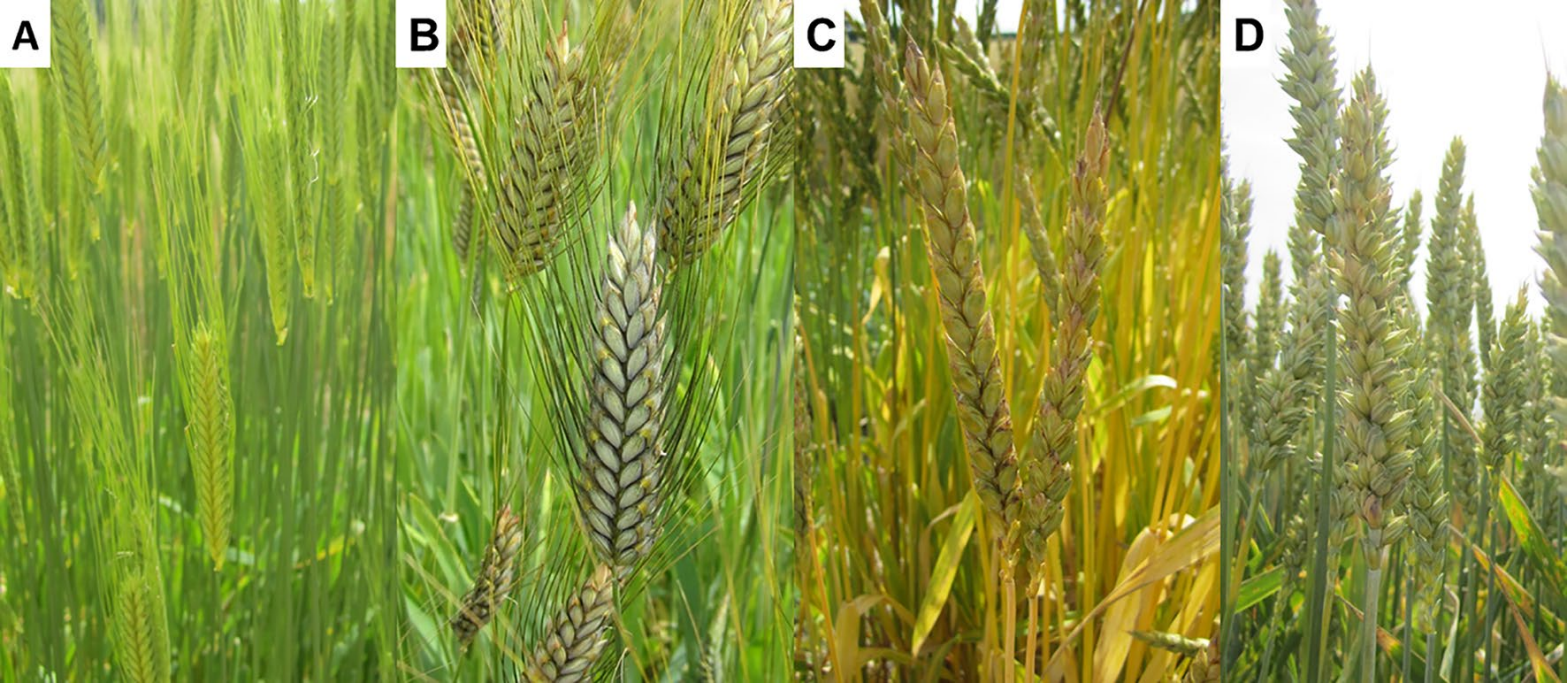
Ears of: A- einkorn, B- emmer, C- spelt and D- modern cultivar of *T. aestivum* L. Photos: S. Geisslitz

Although there is no generally accepted definition of ‘ancient’ wheats, the term is most often applied to three types of wheat which were widely cultivated in former times but, due to relatively low yields, are now only grown at small scale. These are diploid einkorn, tetraploid emmer and hexaploid spelt (Fig. 2). The term ‘heritage’ is most often applied to older types of bread and durum wheats which were grown before intensive plant breeding.

**Fig. 3:**
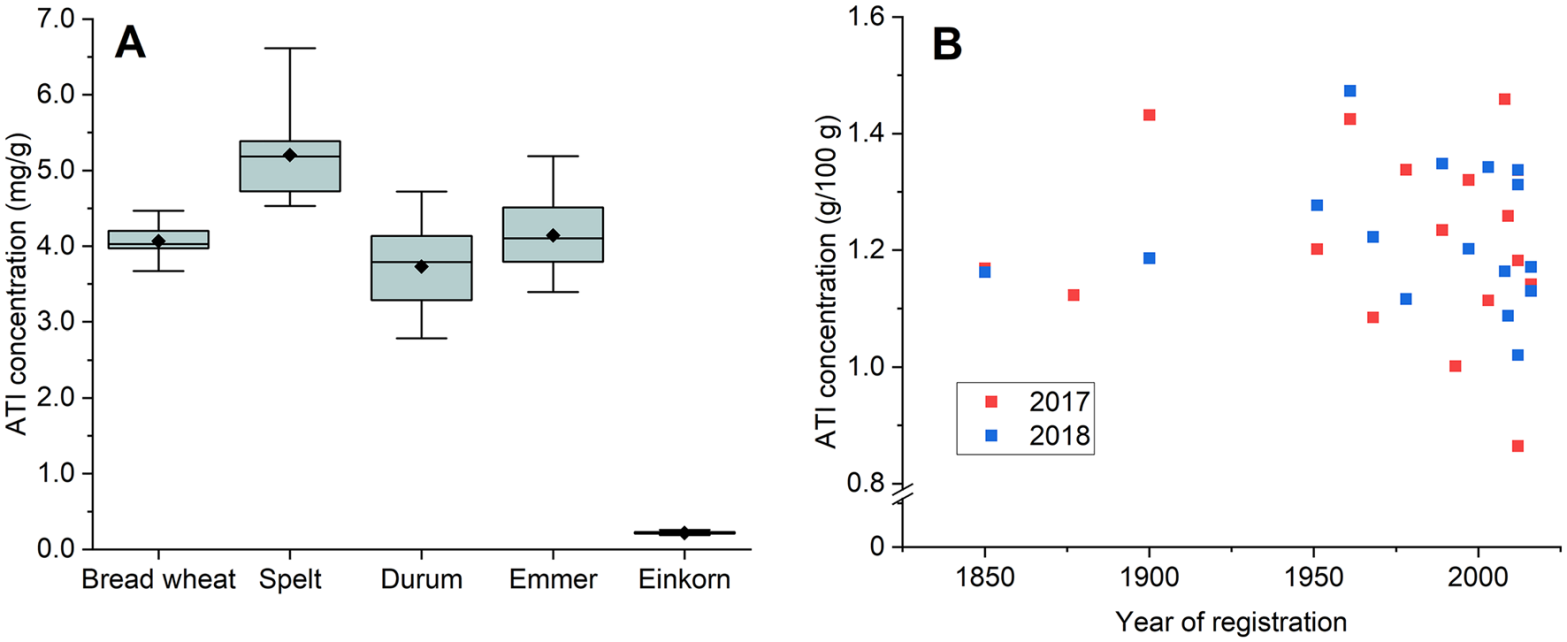
Concentrations of ATIs in different types of wheats. 3A, comparison of modern bread wheat (*Triticum aestivum* L.), durum wheat (*Triticum durum*) and the older types of wheat, spelt (*Triticum spelta*), emmer (*Triticum dicoccon*) and einkorn (*Triticum monococcum*) (modified from Geisslitz et al. [9]). The point in the box is the mean, the line in the box the median, the whiskers show the minimum and maximum and the box to the 25% and 75% percentile. 3B, comparison of bread wheat cultivars according to the year of registration from 1850 to 2010 (red: harvested in 2017 and blue in 2018; modified from Call et al. [8])

A number of studies have compared the amounts and composition of ATIs in different types of wheat with inconclusive results [8–13]. The presence and composition of ATIs is specific for the ploidy level of wheats and can be used to distinguish between diploid, tetraploid and hexaploid wheats [9, 11]. Isoforms called 0.19, CM1 and CM17 are only present in hexaploid wheats, while other isoforms called CM2, CM3 and CM16 are more abundant in tetraploid wheats and 0.19, 0.28 and 0.53 are more abundant in hexaploid wheats. There is also clear evidence that einkorn has lower total concentrations of ATIs [8–11]. Despite these differences, there is wide variation in the concentrations of ATIs within and between the different types of ancient and more modern wheats (Fig. 3A) [9, 11] with no evidence for lower concentrations of ATIs in older types of bread wheat compared to modern high-yielding bread wheats (Fig. 3B) [8, 13]. There is wide variation in the amount of ATIs in bread wheats [12, 13], but the genetic control is inconclusive being reported as high for Australian wheats [12], but low for European wheats [13]. The complex genetic architecture of ATIs indicates that it will be very difficult to select for cultivars with low ATI concentration using traditional breeding methods. Alternatively, genome editing methods (e.g., CRISPR-Cas9) could potentially be more promising techniques [14, 15], but these techniques are currently not accepted for food crops in the European Union.

### Challenges for the analysis of ATIs

Sensitive, precise and reproducible methods are essential to quantify the amounts of ATIs present in plant materials. It is also essential that the grain samples used for comparative studies are well characterised, particularly with respect to the conditions under which the plants were grown, since these will influence grain composition. The first step is to extract the ATIs from the grain (usually flour), using methods which extract all components and are compatible with retaining their structures and activities. However, these methods may also extract other proteins and components, for example, other types of amylase and protease inhibitors that are distinct from ATIs but may interfere with the determination of amount and biological activity (in both in vitro and in vivo studies).

Processed foods, such as dough, bread, pasta and beer, are particularly challenging as they are very complex matrices and the modification and denaturation of proteins may occur during their production. Consequently, the quantitative extraction of biologically active ATIs from such matrices is difficult and, in some cases may not be possible. This must be borne in mind when determining the biological activity of foods as consumed versus the effects of exposure to crude extracts or partially pure fractions, as used in many in vitro and animal studies.

### Effects of ATIs in adverse reactions to wheat

ATIs have been implicated in three types of adverse reaction to wheat (Table 1).

**Table 1:** Overview of the characteristics of adverse reactions to wheat gluten and ATIs, modified from Scherf and Koehler [16]

	Wheat allergy	Celiac disease	NCWS
Prevalence	0.5–4%	1%	0.6–6%
Time until start of symptoms	Minutes–hours	Days–weeks*	Hours
Symptoms	Intra-/extra-intestinal	Intra-/extra-intestinal	Intra-/extra-intestinal
Triggering proteins	Gluten/ ATIs/other wheat proteins	Gluten	ATIs/gluten/other wheat proteins?
Immune response	Adaptive	Adaptive/Innate	Innate
Antibodies	IgE	IgA/IgG subclasses**	IgG subclasses**
Intestinal damage	None	Yes	Probably
Intestinal barrier dysfunction	None	Yes	Probably
Therapy	Wheat-free diet	Gluten-free diet	Wheat- or gluten-free diet

‘Extra-intestinal’ refers to symptoms presenting outside the gastrointestinal tract. *Ig* immunoglobulin, *ATIs* amylase/trypsin inhibitors. *However, coeliac disease symptoms may remain unnoticed/undiagnosed for many years. **A recent study [12] showed that the B cells of coeliac disease patients produced a subclass profile of IgG antibodies (IgG1, IgG3) with a strong inflammatory potential that is linked to autoimmune activity and intestinal cell damage. By contrast, patients with NCWS produced IgG antibodies (IgG4, IgG2) that are associated with a more restrained inflammatory response

### Wheat allergy

Classical food allergy to wheat has a low prevalence and is more common in children who usually grow out of it, leading to an estimated prevalence of 0.1% to 0.3% in adults [17]. Many proteins, including a number of isoforms of ATIs, have been implicated in triggering a response in challenges resulting in high levels of corresponding immunoglobulin E (IgE) [4, 5, 18, 19]. However, ATIs have not been implicated in the most severe allergic response to wheat (anaphylaxis) in which gluten proteins are the major agents [20].

An allergic response to ATIs is a significant issue when flour dust is inhaled and makes contact with tissues within the lungs [2]. This is called bakers’ asthma and is the most prevalent occupational allergy in many countries (affecting, for example, 40% of bakers in the UK). It is a typical airway allergy characterised by raised levels of IgE to a number of proteins, notably ATIs but also including gluten proteins. However, patients with bakers’ asthma can tolerate ingestion of wheat bread [21]. Nevertheless, the fact that in vitro studies indicate that ATIs can induce an innate immune response and may be involved in intestinal reactions (as discussed below) warrants further studies in humans.

### Coeliac disease

Coeliac disease (CD) is the most widespread and well-characterised adverse reaction to gluten consumption. CD is an auto-immune reaction mediated by an inflammatory T cell response. The identification of wheat gluten proteins as the major triggering substances in CD dates from the 1950s and since then, about 40 CD-active peptides (short sequences of amino acids, called “epitopes”, that are recognized by the immune system) have been identified in wheat gluten proteins (gliadins and glutenins) and related proteins from barley and rye [22]. However, a number of other proteins which may play a potentiating role in CD have been identified and Junker et al. [23] and Zevallos et al. [7] have reported evidence for a role of ATIs in the initiation of CD through the induction of inflammation and an innate immune response. Therefore, although it is clear that gluten proteins are the major triggers in CD, the roles of other proteins and triggers (such viral infections) are less clear and require further research.

### Non-coeliac wheat sensitivity (NCWS)

A range of adverse effects which are not due to coeliac disease have been reported after the consumption of wheat products. These include gastrointestinal symptoms, such as bloating and diarrhoea/loose stools, but also wider symptoms, such as tiredness, headache, pain in muscles and joints, depression and anxiety [24]. Although this syndrome was initially defined as ‘non-coeliac gluten sensitivity’ (NCGS) [25], the role of gluten has not been established and it is now more widely referred to as ‘non-coeliac wheat sensitivity’ (NCWS). According to the Salerno criteria [26], the diagnosis for NCWS should include a clinical response to a gluten-free diet as well as a response after a subsequent gluten re-challenge. However, the diagnosis of NCWS is difficult in practice with most patients being self-diagnosed, and the precise prevalence is therefore difficult to determine. Hence, even scientifically based estimates vary widely, from less than 1% to about 10% of the population [24]. Although there is wide overlap in symptoms, diagnostic markers present in wheat allergy (elevated IgE levels) or CD (adverse effects on the structure of the duodenum, presence of certain genetic markers and elevated blood levels of an enzyme involved in allergic reactions) cannot be detected in NCWS, showing that it is a distinct adverse reaction. Some overlap also exists between the symptoms of NCWS and irritable bowel syndrome (IBS).

The mechanisms causing NCWS symptomatology are not completely understood but are likely to be complex, including a role of ATIs which have been shown to activate receptors in the cell membrane, inducing an innate immune response [27]. In addition, sugars which are poorly absorbed in the small intestine but fermented in the colon (referred to as fermentable oligosaccharides, disaccharides, monosaccharides and polyols (FODMAPs)), have been suggested as causative substances [28]. However, the scientific consensus is that although FODMAPs may cause intestinal distress due to gas formation/bloating and osmotic effects/laxation, which are often reported by self-diagnosed NCWS patients, these symptoms are not specific to wheat.

Given the complexity and no clarity of potential causes of NCWS, the role of ATIs is also far from clear and no direct evidence of the impact of ATIs on the gut has been demonstrated with in vivo studies. However, given the impact of ATIs on the lung lining in bakers’ asthma and on the skin in bakers’ eczema, the impact of ATIs on the gut wall (‘the outside inside’), warrants in vivo investigations to clarify their role in NCWS.

Four factors need to be considered in relation to the activities of ATIs in vivo:

1. The total amounts of ATIs present in wheat species and in wheat-based foods.
2. The proportions of the different ATI isoforms, which differ in their biological properties (including allergenicity, enzyme inhibition and immune reactivity).
3. Whether their inhibitory activity (against amylase and proteases) is relevant to their activity in humans, one can speculate that inhibition of amylases can lead to incomplete degradation of starch, resulting in the passage of starch fragments into the colon where they are fermented to gas leading to bloating and abdominal discomfort in susceptible individuals. Similarly, the ability of protease inhibitors to reducing the degradation of proteins could enhance their allergenic potential (see below).
4. Other components that are present in the grain or extracts from grain-based foods and may be responsible for triggering NCWS.

### Effects of processing and digestion on ATIs integrity and bioactivity

Proteins may be modified in various ways during processing including heat-induced chemical modification, such as glycation in Maillard reactions and denaturation, and degradation by endogenous cereal proteases and/or proteases secreted by microbiota during dough fermentation. Such modifications can result in reduced bioactivity, for example, inactivation of enzymes or loss of allergenicity. Alternatively, increased bioactivity may also result, for example, by enhanced exposure of epitopes, which induce an allergic reaction. For example, whereas boiling of eggs reduces allergenicity [29], dry roasting of peanuts can drastically increase allergenicity [30]. Resistances to denaturation and digestion are common properties of allergenic proteins [31] (including ATIs) and are associated with a tightly folded protein structure stabilised by cross-links. Consequently, although the partial proteolysis of native ATIs may not result in complete loss of structure, further degradation during digestion may occur rapidly if the cross-links are broken during processing. It is therefore important to know whether food processing can modify the structures of ATIs and whether this reduces or increases their bioactivity.

Although the effects of food processing on the enzyme inhibitory activity of ATIs are relatively easy to measure, the effects of processing on the effects of ATIs in humans remain unclear. Whereas some studies indicate that ATIs are inactivated and/or degraded during bread baking and the cooking of pasta [32], others indicate that they survive these processes [33, 34]. Furthermore, while ATIs may survive these processes, chemical modification of the structure or changes in the conformation (shape) of the protein may result in loss of inhibitory activity and/or reduced allergenic activity. Conversely, as discussed above, partial digestion or changes in conformation of proteins may expose allergic parts of the protein resulting in increased severity of reactions.

It has also been shown that specific lactobacilli present in sourdough bread making systems secrete enzymes which degrade ATIs [35–38] and, may either increase or decrease their bioactivity, depending on the patterns and the degree of the degradation.

Finally, it is important to note that some food products that contain wheat grain or flour have not been subjected to food processing for example raw cookie dough, unprocessed flaked wheat as used in muesli and dusting flour used to facilitate dough handling and for bread decoration. There will therefore be less impact of processing on the structure or bioactivity of ATIs in these materials resulting in potentially stronger health effects.

It is clear that the effects of food processing on the bioactivity of ATIs are complex and are also likely to differ between isoforms of ATI. It is therefore important to determine whether some isoforms of ATIs are more sensitive to different processing systems than others. Because quantifying ATIs in various food matrices is very challenging, it is also important to improve analytical procedures. This knowledge may allow us to tailor the processing conditions for specific types of wheat. It may also enable us to identify types of wheat with low contents of the most active ATI isoforms and select for low activity in future wheat breeding programmes.

However, above all, it is essential that the assumed impacts of ATIs on health are validated by data obtained from human studies. In vitro studies carried out with isolated protein fractions or animal model studies are not sufficient to provide evidence on in vivo effects in humans consuming processed wheat-based foods.

## Conclusion

Wheat is one of the most important staple foods and it has been cultivated and consumed for millennia. The nutrient content and safety of wheat are therefore crucial for food security. Over 90% of the population can eat wheat products without adverse effects but small proportions suffer from allergy, CD or NCWS. ATIs are present in cultivated and wild wheat species and cereals and have well-documented roles in wheat allergy (bakers’ asthma and food allergy). I*n vitro* studies and in vivo studies in animal models have shown that they also play a role in the pathogenesis of CD and NCWS, but many questions remain unanswered. The relative activities of different ATI isoforms from different sources, such as different types and cultivars of wheat, on humans including the mechanism at the molecular level are still not clear. Possible approaches to reduce ATIs and their bioactivity include food processing and plant gene editing. Further information on the impacts of ATIs on human health during the whole chain from cultivation over processing to consumption is required to underpin these improvements.
